# Educational Attainment: A Genome Wide Association Study in 9538 Australians

**DOI:** 10.1371/journal.pone.0020128

**Published:** 2011-06-09

**Authors:** Nicolas W. Martin, Sarah E. Medland, Karin J. H. Verweij, S. Hong Lee, Dale R. Nyholt, Pamela A. Madden, Andrew C. Heath, Grant W. Montgomery, Margaret J. Wright, Nicholas G. Martin

**Affiliations:** 1 Genetic Epidemiology Laboratory, Queensland Institute of Medical Research, Brisbane, Australia; 2 School of Psychology, University of Queensland, Brisbane, Australia; 3 School of Medicine, Washington University, St Louis, Missouri, United States of America; Tor Vergata University of Rome, Italy

## Abstract

**Background:**

Correlations between Educational Attainment (EA) and measures of cognitive performance are as high as 0.8. This makes EA an attractive alternative phenotype for studies wishing to map genes affecting cognition due to the ease of collecting EA data compared to other cognitive phenotypes such as IQ.

**Methodology:**

In an Australian family sample of 9538 individuals we performed a genome-wide association scan (GWAS) using the imputed genotypes of ∼2.4 million single nucleotide polymorphisms (SNP) for a 6-point scale measure of EA. Top hits were checked for replication in an independent sample of 968 individuals. A gene-based test of association was then applied to the GWAS results. Additionally we performed prediction analyses using the GWAS results from our discovery sample to assess the percentage of EA and full scale IQ variance explained by the predicted scores.

**Results:**

The best SNP fell short of having a genome-wide significant p-value (p = 9.77×10^−7^). In our independent replication sample six SNPs among the top 50 hits pruned for linkage disequilibrium (r^2^<0.8) had a p-value<0.05 but only one of these SNPs survived correction for multiple testing - rs7106258 (p = 9.7*10^−4^) located in an intergenic region of chromosome 11q14.1. The gene based test results were non-significant and our prediction analyses show that the predicted scores explained little variance in EA in our replication sample.

**Conclusion:**

While we have identified a polymorphism chromosome 11q14.1 associated with EA, further replication is warranted. Overall, the absence of genome-wide significant p-values in our large discovery sample confirmed the high polygenic architecture of EA. Only the assembly of large samples or meta-analytic efforts will be able to assess the implication of common DNA polymorphisms in the etiology of EA.

## Introduction

Access to education is considered to be a predictor for a wide range of later life outcomes such as employment [1], income [2], and health outcomes such as obesity [3], [4]. In addition to its relevance to economics and health, educational attainment (EA) is a measure of interest in the study of cognitive abilities/intelligence. The correlation between EA and measures of cognitive ability ranges from 0.45 up to 0.80 [5], [6], [7]. Three hypotheses of causality have been postulated to explain the underlying mechanisms of the relationship between EA and cognitive functioning, (i) intelligence/cognitive abilities are a cause of EA because intelligence is believed to be more biologically anchored than scholastic achievements [8], (ii) cognitive abilities are a product of scholastic achievement [9], [10] and (iii) basic cognitive processes such as reaction time, inspection time and memory recall partly determine both scholastic achievement and cognitive performance [11]. At present, none of these hypotheses have been discarded and it is likely that a mixture of all three plays a role in the link between EA and cognitive abilities. Additionally, a recent report suggested that the causal relationship between EA and intelligence varies according to an individual's level of intelligence [7].

Biological processes involved in scholastic achievement and cognitive performance are likely to be shared to some extent. Twin studies that take advantage of differences between genetically identical twins (monozygotic or MZ) and fraternal twins (dizygotic or DZ), have shown that EA and cognitive performance are influenced to a large extent by common genetic factors [12], [13], [14] and the heritability of EA has been estimated as high as 80% [6], [15]. Therefore, investigating the biological etiology of educational attainment could provide insights into the molecular basis of cognition because of its strong heritability and high correlation with cognitive performance. Moreover, EA has the advantage of being a measure that is much easier to collect than IQ and therefore it is more viable to assemble the large cohorts necessary to perform genome wide association scans (GWAS).

Molecular genetics studies of EA have been largely limited to a candidate gene approach including the Catechol-O-methyltransferase (*COMT*) [16], [17], the Brain-derived neurotrophic factor (*BDNF*) [18] and the Dopamine receptor D4 (*DRD4*) [19]. The choice of these genes as candidates for EA is strongly hypothesis driven due to their characterized neurobiological functions but convincing replications are lacking in support of their hypothetical role in the etiology of EA. The advances in microarray technologies in recent years have made it possible to genotype millions of single nucleotide polymorphisms (SNPs) for a low cost (about US$500 per individual). This has led to a major shift toward a hypothesis free genome-wide association study design. A lot of GWAS studies has emerged in the literature, identifying hundreds of polymorphisms and genes associated with complex traits and diseases. In this study we present the results of a GWAS for EA, using an Australian twin family discovery sample of 9538 individuals from the general population and a further 968 individuals as an independent replication sample.

## Methods

### Participants

The participants for our discovery sample were drawn from two cohorts of adult twin families (cohorts 1 and 2) that have taken part in a wide range of studies of health and well-being. Individuals from Cohort 1 were born before 1964 and individuals from cohort 2 were born between 1964 and 1981. Both these cohorts have participated in previous postal questionnaire and telephone interview studies, and recruitment was extended to their relatives (parents, siblings, adult children and spouses). Our total discovery sample was composed of 9538 individuals for whom both EA and genome-wide SNP genotype data were available. Our replication sample (Cohort 3) consisted of 968 individuals who are the parents of adolescent twins participating in our melanoma risk factors and cognition studies (1996-ongoing) [20]. Information for the different studies is available in Table S1.

For the prediction analysis of cognitive ability based on the EA GWAS results obtained in the discovery sample, we used one of the twin children of cohort 3. Their full scale IQ (FSIQ) was collected as part of the cognition study and genotyping data were available for 1842 adolescent twins and their siblings ranging in age from 15 to 22 years (mean = 16.28 years ±0.45 SD).

Ethical approval, for the studies from which the data drawn, was obtained from the Human Research Ethics Committee of the Queensland Institute of Medical Research. Informed written consent for all measures was obtained from each participant and their parents/guardian if participants were younger than 18 years of age.

### Educational attainment

**Figure 1:**
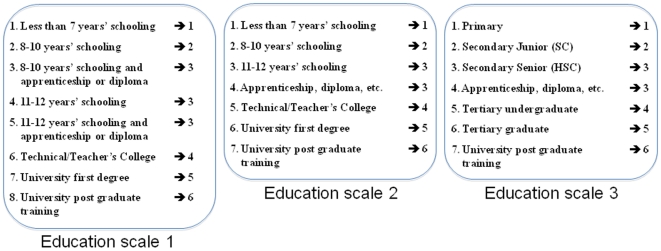
Transformation applied to the original education scales (1 to 3) to obtain a new harmonized scale across studies.

Self reported educational attainment (EA) was collected as part as of questionnaires and telephone interviews. In the adult cohorts (1 and 2) three similar education scales were used to collect EA depending on the study in which an individual participated (fig. 1). Each individual score was transformed to create a new 6-level EA scale harmonised across the studies, with 1 = 7 years or less of schooling, 2 = 8 to 10 years of schooling, 3 = 8 to 10 years of schooling + apprenticeship or 11 to 12 years of schooling or 12 years of schooling + apprenticeship, 4 = teacher college or technical college, 5 = university undergraduate training and 6 = university postgraduate training (fig. 1). For a number of individuals (n = 5314) multiple reports of EA were available and the highest education level reported was selected for analysis. In cohort 3, EA was also assessed as part of a questionnaire that the parents of the adolescent twins answered while their children underwent cognitive testing. EA was recorded with one of the scales previously used in the adult cohorts (Figure 1). All individuals that were included in the GWAS analysis were at least 21 years of age (the standard age of first degree graduation). Descriptive statistics of the age and educational attainment of the participants according to their study of origin can be found in Table S1.

### Cognitive abilities

Cognitive abilities were measured by full scale IQ (FSIQ) that was assessed using the Multi-dimensional Aptitude Battery (MAB) [21]. The MAB is a general intelligence test designed to mirror the WAIS-R [22] and it is presented in a multiple-choice format. Participants completed three verbal (information, arithmetic, vocabulary) and two performance subtests (spatial, object assembly) which were combined here to form a full-scale IQ score. Twins completed the MAB as close as possible to their 16th birthday when they came to participate in the cognition study. Further details of the IQ testing procedure have been previously published [23], [24].

### Genotyping and Imputation

The genotypic data used in the current study come from a large genotyping project involving nine waves of genotyping that used three different Illumina SNP chips (Human610-Quad, HumanCNV370-Quadv3 and Human 317K) [25]. For each genotyping wave, rigorous quality control (QC) steps were applied to ensure the highest standard of the pre imputation data. Details of the QC steps have been described elsewhere [25]. Imputation of the autosomal chromosomes was performed in two stages with MACH [26] using a set of SNPs (N = 269840) common to the different Illumina chips. These data were screened for ancestry outliers. Full siblings and offspring of individuals who had been identified as ancestry outliers were excluded from the reference set used in MACH stage 1. In the first stage, the data from a set of 450 reference individuals from our set were compared to the phased haplotype data from the HapMap samples of european ancestry (CEU I+II) (release 22, build 36). These 450 reference individuals were made up of fifty unrelated individuals (with the lowest missingness) from each of the nine subsamples. Stage 1 generated recombination and error files that describe how our data relate to the HapMap data, in effect allowing us to customise the HapMap data for our population. In the second stage, data were imputed for the 17,862 individuals using the HapMap data (release 22, build 36) as the reference panel and the recombination and error files generated in stage 1 to customise the imputation. SNPs with a minor allele frequency <.01, SNPs with an Rsq imputation quality score <.3 were excluded. A panel of 2,480,163 autosomal SNPs and a panel of 13783 genotyped X-chromosome SNPs were use for association analysis.

### Data analysis

Genome wide association analysis using dosage scores was performed in MERLIN offline [27] to account for family structure. The association analysis of genotyped markers on the X-chromosome were conducted in Minx (as implemented in MERLIN). Sex, year of birth (YOB), age, YOB×sex, YOB^2^, YOB^2^×sex, age^2^, Age×sex and Age^2^×Sex were used as covariates. Both YOB and age were used as covariates in this study: YOB in order to account for changes in terms of access to education that have occurred since the early 1900's and age in order to account for the fact that the older an individual is, the less likely he/she is to undertake further education. Visualisation and annotation of the GWAS results was conducted using WGAViewer [28] and the pruning for linkage desquilibrium (LD) (r^2^<0.8) of the top 200 SNPs was performed in SNAP [29] based on the HapMap release 22 CEU panel.

Additionally, The VEGAS gene-based test [30] that can be used with related individuals was performed using the GWAS output data of our discovery sample. The test summarizes evidence for association on a per gene basis by considering the full set of SNPs within the gene (determined by SNPs lying within ±50 kb of a gene's 5′ and 3′ UTRs) and the LD between them.

### Power analyses

It is expected that many genes of very small effect size contribute to the genetic variance of complex traits. We estimated the empirical power our discovery sample provides to detect genetic variants explaining 1%, 0.5% and 0.2% of the phenotypic variance by running association tests on simulated datasets in Merlin. The simulated datasets are similar to the original data in terms of marker informativeness, spacing, allele frequency, trait distribution, and missing data patterns, but they are simulated such that a selected SNP accounts for a specified proportion of the variance. The selected SNP had minor allele frequency 0.25. Association analysis was conducted on 1000 data sets generated by the simulation procedure. The empirical power is estimated as that proportion of the 1000 association analyses in which a genome-wide significant association (α = 5*10^−8^) was detected. Results indicated that our sample provides 100%, 80% (799 out of the 1000 simulations) and 13% (130 out of the 1000 simulations) power to detect SNPs that explain 1%, 0.5% and 0.2% of the variance in EA, respectively.

### Prediction analyses

Prediction analyses were conducted in two stages. In stage 1, the effect sizes of the 2,480,163 SNPs from GWAS for EA, as well as 5 sub-panels of SNPs (based on p-value thresholds of p<0.5, p<0.4, p<0.3, p<0.2 and p<0.1), were extracted from the MERLIN [27] output of our discovery sample. Based on the effect sizes for these panels and the imputed SNP data of our adolescent twin families we generated 6 sets of prediction scores using the PLINK [31] scoring routine for cohort 3 (968 parents and only one of their twin children (n = 799) in order to have a sample of independent individuals). In stage 2, we compared the predicted scores of the parents to their EA by fitting EA as a function of the predicted scores in a linear model (EA∼predicted scores) using R (http://cran.r-project.org/) to evaluate to percentage of variance explained (R^2^) by the predicted score and its level of significance. Similarly, we fitted the FSIQ of one of the twin children as a function of their predicted scores in a linear model (FSIQ∼predicted scores).

## Results

**Figure 2:**
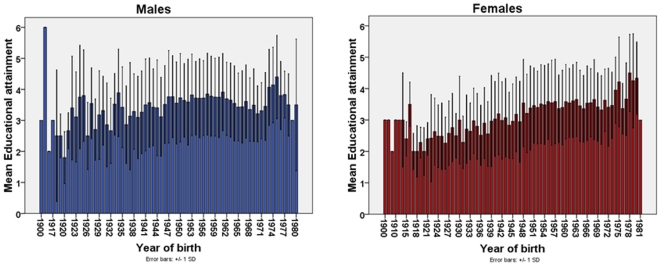
Mean educational attainment (EA) by year of birth in male and female.

We examined educational attainment for over 9538 individuals born between 1900 and 1981 from 3764 families. A similar distribution of the mean educational attainment was observed between males and females (figure 2). We observed a gradual increase of the mean EA from 8–10 years of schooling to above 11 to 12 years of schooling for individuals born during the first half of the 20^th^ century (Figure 2). The mean EA of individuals born between the late 40's up to the mid 70's stayed constant around 3.5 before increasing close to 4 (4 = teacher college or technical college) for individuals born after the early to mid 70's (figure 2).

Using DNA previously collected for these individuals we performed a GWAS for EA. The heritability of EA was estimated 63.5% by MERLIN and is comparable to the 57% previously reported in our earlier variance components analysis of EA in cohort 1 [6]. Table 1 reports the results for the top 50 most associated SNPs after pruning for LD (r^2^<0.8), with the best-associated SNP rs2680324 having a p-value of 9.77×10^−7^ (the Q-Q plot is presented in Figure S1 and the manhattan plot in Figure S2). We then tested the association of these 50 SNPs with EA in an independent replication sample of 968 individuals. Five of these SNPs had an association p = value below 0.05 and an effect in the same direction: rs226039 (p = 0.040), rs976928 (p = 0.0036), rs4298928 (p = 0.004), rs7106258 (p = 0.00097) and rs226047 (p = 0.039). However, only rs7106258 survived correction for multiple testing (p = 0.05/50 = 0.001).

**Table 1:** Top 50 most associated SNPs in 9538 individuals after pruning for LD (r^2^<0.8).

rank	SNP	Chr.	Position in bp	SNP type	Closest gene	*p*-value	SNP in LD*
1	rs2680324	10	56970071	INTRONIC	PCDH15	9.77E-07	2
2	rs10079475	5	179585240	INTRONIC	RASGEF1C	1.36E-06	3
3	rs8085689	18	21656634	INTRONIC	TTC39C	5.20E-06	2
4	rs13092348	3	116580097	INTRONIC	LSAMP	5.40E-06	17
5	rs1521417	3	116761113	INTRONIC	LSAMP	5.55E-06	2
6	rs11004778	10	56974190	INTRONIC	PCDH15	5.58E-06	9
7	rs2246477	5	172208454	UPSTREAM	Y_RNA.479	7.27E-06	1
8	rs914925	9	93584793	INTRONIC	SYK	8.16E-06	2
9	rs226039	16	21288101	INTRONIC	CRYM;	8.98E-06	0
10	rs976928	11	81479043	INTERGENIC	RP11-179A16.1	9.60E-06	6
11	rs7191909	16	21262897	3PRIME UTR	ANKS4B	1.02E-05	15
12	rs4298928	11	81470414	INTERGENIC	RP11-179A16.1	1.11E-05	0
13	rs6767669	3	116599500	INTRONIC	LSAMP	1.19E-05	0
14	rs6581191	12	58875698	INTERGENIC	RP11-652N17.1	1.23E-05	0
15	rs7281948	21	32970928	INTERGENIC	FBXW11P1	1.23E-05	2
16	rs1517635	2	224203115	INTERGENIC	AC068035.1	1.32E-05	0
17	rs8082313	17	17976932	INTERGENIC	C17orf39	1.40E-05	0
18	rs2431664	5	172203911	WITHIN NON CODING GENE	RP11-779O18.3	1.50E-05	0
19	rs17157509	7	109881775	INTERGENIC	AC003088.1	1.52E-05	6
20	rs12497655	3	116764010	INTRONIC	LSAMP	1.82E-05	1
21	rs13402289	2	100659389	INTRONIC	AFF3	1.89E-05	6
22	rs7106258	11	81484019	INTERGENIC	RP11-179A16.1	1.92E-05	14
23	rs10068846	5	179588797	INTRONIC	RASGEF1C	2.01E-05	0
24	rs5009524	20	38894887	INTERGENIC	RP1-191L6.2	2.17E-05	0
25	rs226047	16	21301472	INTRONIC	CRYM	2.30E-05	3
26	rs744684	7	154196001	INTRONIC	DPP6	2.32E-05	0
27	rs226038	16	21288338	INTRONIC	CRYM	2.37E-05	0
28	rs6691053	1	173868955	DOWNSTREAM	ZBTB37	2.37E-05	0
29	rs1011689	10	97760476	INTRONIC	CC2D2B	2.38E-05	6
30	rs4876153	8	2304334	INTERGENIC	AC133633.2	2.38E-05	0
31	rs7672521	4	175302173	UPSTREAM	RP11-51M24.1	2.58E-05	0
32	rs2029238	18	21702106	INTRONIC	AC090772.1	2.63E-05	2
33	rs9903961	17	10732872	UPSTREAM	PIRT	2.75E-05	0
34	rs1122208	15	58623525	INTERGENIC	LIPC	2.82E-05	3
35	rs11194701	10	111292456	UPSTREAM	BTF3P15	2.92E-05	0
36	rs2297679	1	32157009	INTRONIC	COL16A1	3.00E-05	1
37	rs2044541	11	41343185	INTRONIC	LRRC4C	3.36E-05	1
38	rs1793791	11	131111013	WITHIN NON CODING GENE	AP002856.7	3.61E-05	0
39	rs6011899	20	61356257	INTRONIC	NTSR1	3.76E-05	0
40	rs472975	1	185534476	WITHIN NON CODING GENE	GS1-204I12.2	3.83E-05	12
41	rs256940	5	114866191	INTRONIC	FEM1C	3.84E-05	0
42	rs4514783	18	61918306	INTERGENIC	AC100848.1	3.85E-05	4
43	rs11876820	18	61891408	INTERGENIC	AC100848.1	4.12E-05	0
44	rs2275998	11	66326581	INTRONIC	CTD-3074O7.2	4.15E-05	0
45	rs7936167	11	40156059	INTRONIC	LRRC4C	4.40E-05	4
46	rs6986402	8	17630589	INTRONIC	MTUS1	4.43E-05	1
47	rs10057387	5	172230003	INTERGENIC	RP11-536N17.1	4.49E-05	0
48	rs6938281	6	25312488	INTRONIC	LRRC16A	4.54E-05	0
49	rs2257055	9	130311404	INTRONIC	FAM129B	4.57E-05	0
50	rs6584475	10	103806793	INTRONIC	C10orf76	4.68E-05	1

*number of SNPs in LD (r2≥0.8) among the top 200 GWAS hits.

To completely utilise the GWAS results, we then performed a gene-based test that maps SNPs to their respective genes if they are located within 50 kb of a gene locus. The gene-based test produced an empirical p-value for 17549 autosomal genes. The top 50 most associated genes are reported in table 2 and the best result was obtained for FLJ41766 (p = 1.4×10^−5^), however, it does not reach the multiple-testing correction threshold (p = 0.05/17549 = 2.8*10^−6^).

**Table 2:** Top 50 most associated genes with the EA in 9538 individuals.

rank	Chr.	Gene	Number of SNPs	Start position	Stop position	P-value
1	16	FLJ41766	35	21219670	21237413	1.40E-05
2	16	CRYM	64	21177342	21221918	2.10E-05
3	16	ANKS4B	82	21152516	21171251	2.80E-05
4	10	NPM3	24	103531071	103533148	6.10E-05
5	10	FGF8	22	103519876	103525817	8.80E-05
6	10	MGEA5	31	103534198	103568165	0.000117
7	14	LTB4R2	110	23848000	23850798	0.000154
8	10	HPS6	12	103815136	103817783	0.000155
9	14	C14orf21	115	23838937	23844214	0.000171
10	14	ADCY4	122	23857409	23873704	0.000175
11	19	ZNF100	83	21698682	21742270	0.000232
12	14	RABGGTA	76	23804583	23810643	0.000237
13	5	DUSP1	108	172127706	172130809	0.000249
14	10	CC2D2B	80	97749872	97782431	0.000263
15	14	TGM1	80	23788159	23802256	0.000319
16	10	KCNIP2	29	103575720	103593667	0.000538
17	1	ZBTB37	53	172104115	172122397	0.000553
18	1	DARS2	45	172060580	172094305	0.000617
19	18	CABYR	76	19972952	19995562	0.000629
20	10	CCNJ	84	97793143	97810612	0.000723
21	17	DYNLL2	49	53515797	53521810	0.000783
22	11	FLRT1	99	63627937	63643221	0.000844
23	10	C10orf76	89	103595345	103805922	0.000847
24	7	C7orf59	44	99584465	99589769	0.000872
25	11	BBS1	64	66034694	66057660	0.000953
26	19	ZNF99	88	22730846	22744624	0.000961
27	11	ACTN3	56	66070966	66087373	0.001017
28	7	C7orf43	41	99589978	99594238	0.00102
29	17	CCDC43	45	40110330	40122691	0.001079
30	11	CTSF	50	66087510	66092623	0.001108
31	14	NFATC4	104	23907093	23918650	0.001127
32	11	DPP3	54	66004455	66033706	0.00114
33	11	ZDHHC24	57	66063310	66070247	0.00117
34	15	RHCG	89	87815643	87840803	0.001346
35	3	APEH	52	49686438	49695938	0.00135
36	7	GAL3ST4	52	99594800	99604309	0.001364
37	1	SERPINC1	57	172139564	172153096	0.001451
38	7	BET1	79	93458935	93471626	0.001466
39	3	RHOA	51	49371582	49424530	0.001497
40	17	SPHK1	52	71892296	71895536	0.001549
41	3	MST1	48	49696391	49701099	0.001573
42	18	CD226	199	65681172	65775212	0.00158
43	3	RNF123	53	49701993	49733966	0.00163
44	5	C5orf39	57	43074938	43076098	0.00166
45	11	PELI3	39	65990911	66001384	0.001683
46	6	LY6G6F	110	31782662	31786351	0.00177
47	3	BSN	87	49566925	49683986	0.001818
48	7	STAG3	50	99613473	99649946	0.00182
49	6	BAT5	131	31762714	31779067	0.00186
50	6	LY6G6C	97	31794403	31797489	0.00188

Our Prediction analyses of EA in our replication sample using predicted scores for different thresholds of GWAS significance (from p<0.1 to the whole genome) showed a regression r^2^ ranging between 0.0011 to 0.0023 (non-significant: p≥0.14) between the predicted scores and EA in the independent sample of adults in all 6 analyses. Similarly, in the children of these individuals (n = 799) when we attempted to predict FSIQ as a function of the predicted scores across the different thresholds of GWAS significance the regression r^2^ were non-significant (p≥0.15) and varied between 9.0*10^−5^ to 0.0026.

## Discussion

In the current study we observed that Educational attainment (EA) has increased constantly during the 20^th^ century from a mean EA of 8–10 years of schooling to around 12 years of schooling followed by apprenticeship, teacher college or technical college. This trend was the same for males and females. However, it should be noted that only 2% of our sample was born before 1926 and 31% before 1951. Thus the data are too sparse to comment on the changes of mean EA in relation to time specific events such as World War II. The majority of our sample was born post World War II and the mean EA for these individuals confirms that pursuing a tertiary education was much more common for these generations than it was for the previous generations. The mean EA for individuals born in this period remained fairly constant and major events such as the Vietnam War (Australian conscription: 1965–1975) or free access to university education (1974–1988) do not appear to have affected the level of EA in our sample.

Using SNP genotyping data for these individuals, we performed a large GWAS study for EA. The strength of our study was its large sample size that conferred 100% and 80% power to detect polymorphisms (MAF = 0.25) explaining 1% and 0.5% of the phenotypic variance respectively. However, despite a good power, no SNP had a p-value significant at the genome wide level of 5×10^−8^. Therefore, we tested the robustness of the top 50 most associated SNPs pruned for LD (r^2^<0.8) in a replication sample. Five of these 50 SNPs reached a p-value smaller than p = 0.05 and an effect in the same direction. Among these SNPs only one had a p-value surviving multiple correction, rs7106258 (p = 0.00097) located in an intergenic region of chromosome 11q14.1 (combined p-value was 3.71*10^−7^). This region deserves future attention as it has been suggested to be involved in other brain phenotypes. This region contains the GAB2 gene, a candidate gene for Alzheimer's disease [32] and copy number variations in this region have also been linked with mental retardation [33].

Overall our GWAS findings are comparable to the latest GWAS report for general cognitive abilities in that no SNPs survived multiple testing correction for genome wide significance [34]. The estimated heritability of EA in our sample was 63.2% and comparable to the 57% found in our behavior genetic analysis of EA [6]. However, we also know from our previous study that the heritability of EA could be as high as 82% in Australia once corrected for assortative mating [6]. So why can we not find SNPs with genome wide significant p-values for a highly heritable trait such as EA? Some elements of an answer can be drawn from what we have learned from other human complex traits such as IQ [34] and height [35] in which common variants of large effect size were not found. Common explanations for the missing phenotypic variance due to genetic variation might be (i) a large number of common variants of small effect sizes (<1%), (ii) rare variants of large effect sizes, (iii) structural variants or (iv) low power to detect epistasis or gene-environment interactions [36]. These questions are being addressed with the formation of a new consortium and meta-analytical efforts that assemble large samples to examine if a large number of common variants of small effect sizes contribute to the current lack of robust association signals. Some new elements of an answer to the above question are already available with Yang and colleagues [37] recently showing that 45% of variance for human height can be explained when all SNP effects from a panel of nearly 300 000 SNPs were considered simultaneously. On the other hand, the potential role of rare and structural variants in the etiology of EA and other complex traits may be answered by the next-generation of association studies [38]. An example of an innovative study design was recently described by Holm et al [39] and was successful to detect low frequency SNPs associated with sick sinus syndrome. Their multi step approach consisted of a classic GWAS followed by whole genome sequencing for a small number of cases and controls, before imputing the newly discovered variants into their original sample. Although the above example was applied to a case-control setting this could be adapted to quantitative traits by sequencing a small number of individuals at the extremes of the distribution.

Another noticeable feature of our GWAS results is that none of the top 50 SNPs fall into exons. This is not surprising as more than 80% of associated variants detected by GWAS were found in non-coding regions [36] which further supports the importance of including these regions in gene mapping studies of complex traits [40].

Moving from a traditional single marker analysis, we performed the VEGAS gene-based test [30]. This is an attractive approach that gives a second life to GWAS data and it might ease the current frustration of the millions of dollars spent in large genotyping projects that cannot produce replicated associations. *FLJ 41766* was the best hit, however, there is no evidence for this gene to suggest a role in neurobiology. A similar observation was found when looking at the genes among the top hits one at the time. Additionally, both the gene-based test and GWAS showed no evidence of association for BDNF (rank = 16132 out of 17549 genes tested by VEGAS), COMT (rank = 197), and DRD4 (rank = 17451), which have been previously associated with EA [16], [17], [18], [19]. However, these candidate gene studies were conducted on small samples that may have not produced genuine association signals. If true, these association signals previously reported should have been detected in our large sample (>9 500 individuals).

One of the major goals of genetic epidemiologists is to identify genetic variants that can be used to predict complex traits and susceptibility risk to diseases. So far, this has been harder than originally thought due to the difficulty of mapping loci of small effect that reflect the highly polygenic architecture of common diseases and complex traits. One recent approach that has been used in this exercise is to generate a predicted score based on a genome wide profile of SNPs effects [41], [42]. Here, we generated an EA predicted score based on different level of p-values obtained in the GWAS of our large discovery sample to see whether or not we could predict EA in an independent sample. The EA variance explained by the six sets of predicted scores was low and non significant. When a similar prediction analysis was performed with FSIQ instead of EA, this percentage of variance was lower than with EA as expected and also non-significant. These low correlations between the predicted phenotypes scores might arise from a low accuracy of the genome wide SNP effects that were used to generate these scores. In the future, it will be most interesting to see the evolution of these results if these analyses were to be repeated once genome wide SNP effects from a larger sample or from a meta-analysis are available.

In the present study we performed one of the largest genome wide association scans to examine the molecular genetics of educational attainment. Despite our large discovery sample and good genome coverage of the genome, no SNP reached genome wide evidence of association. As for many other complex traits (e.g. Human heights [43]), our results confirmed the high polygenic architecture of EA. Future large consortiums combined with sequencing efforts will hopefully bring more insights into the molecular architecture of EA and shed light on whether it is common variants of small effects or rare variants of large effect that contribute to the biological blue print of EA.

## Supporting Information

Figure S1 Quantile-Quantile plot from the GWAS result of educational attainment in 9538 individuals. (Lambda = 1.0229). The grey shade area represents the 95% confidence intervals.
(TIFF)

Figure S2 Manhattan plot of GWAS for educational attainment for 9538 individuals.
X-axis represents the chromosomal location for each SNPs, and Y-axis the −log10 P-value for association with educational attainment.
(TIF)

Table S1 Descriptive statistics for the different cohorts from which the educational attainment data originated.
(DOC)
