# Supplementary figures and images for: Educational Attainment: A Genome Wide Association Study in 9538 Australians
Source: PLoS One. 2011 Jun 9;6(6):e20128. doi: 10.1371/journal.pone.0020128 (PMC3111411; doi:10.1371/journal.pone.0020128)

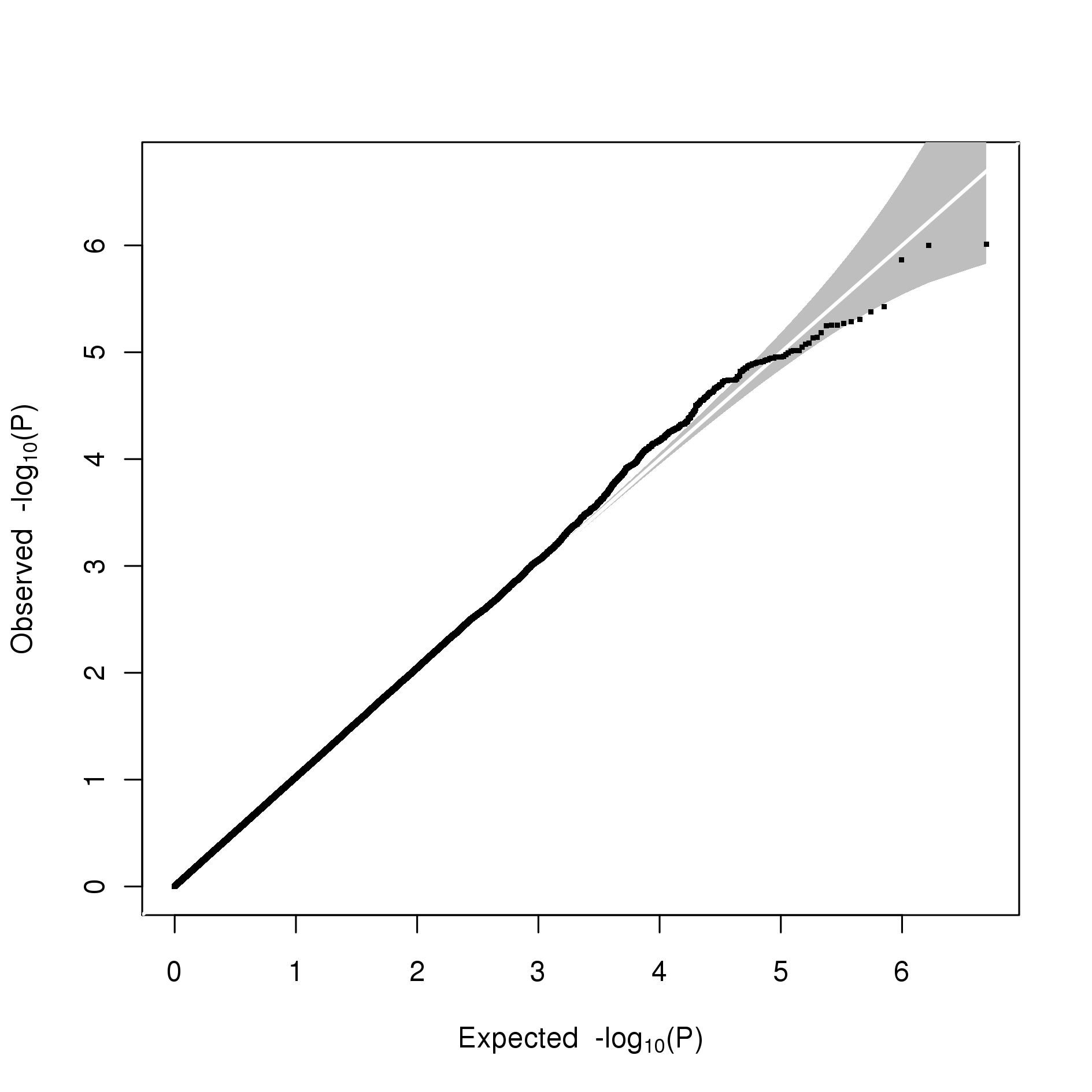

Supplement: Figure S1 — Quantile-Quantile plot from the GWAS result of educational attainment in 9538 individuals. (Lambda = 1.0229). The grey shade area represents the 95% confidence intervals. (TIFF) [file pone.0020128.s001.tiff]

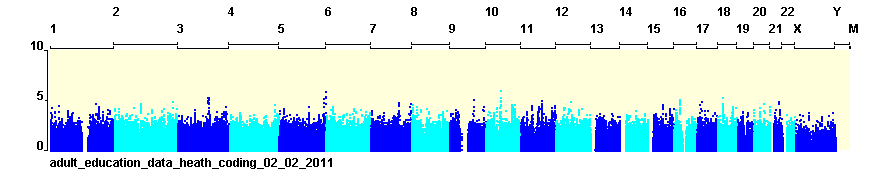

Supplement: Figure S2 — Manhattan plot of GWAS for educational attainment for 9538 individuals. X-axis represents the chromosomal location for each SNPs, and Y-axis the −log10 P-value for association with educational attainment. (TIF) [file pone.0020128.s002.tif]
