# Supplementary material for: Educational Attainment: A Genome Wide Association Study in 9538 Australians
Source: PLoS One. 2011 Jun 9;6(6):e20128. doi: 10.1371/journal.pone.0020128 (PMC3111411; doi:10.1371/journal.pone.0020128)
Supplement: Table S1 — Descriptive statistics for the different cohorts from which the educational attainment data originated. (DOC) [file pone.0020128.s003.doc]

**Table S1.** Descriptive statistics for the different cohorts from which the educational attainment data originated

|  |  |  |  | Age | | Harmonised EA scale | | | |
| --- | --- | --- | --- | --- | --- | --- | --- | --- | --- |
| Cohort | Year of EA data collection | Illumina SNP platform | N | Mean (SD) | Range | | Male  mean (SD) | Female mean (SD) | Range |
| Cohort 1 | 1979-ongoing | CNV370K, 317K & 610K | 6864 | 44.2 (11.4) | 21-101 | | 3.6 (1.2) | 3.3 (1.2) | 1-6 |
| Cohort 2 | 1989-ongoing | CNV370K, 317K & 610K | 2674 | 30.2 (4.9) | 21-40 | | 3.5 (1.1) | 3.5 (1.1) | 1-6 |
| Cohort 3 | 1996-ongoing | 610K | 968 | 47.5 (5.1) | 32-77 | | 3.3 (1.3) | 3.2 (1.2) | 1-6 |
|  |  | TOTAL | 10506 | 41 (11.5) | 21-101 | | 3.5 (1.3) | 3.3 (1.2) | 1-6 |
